# Spatial epidemiology of dry eye disease: findings from South Korea

**DOI:** 10.1186/1476-072X-13-31

**Published:** 2014-08-15

**Authors:** Sun-Bi Um, Na Hyun Kim, Hyung Keun Lee, Jong Suk Song, Hyeon Chang Kim

**Affiliations:** 1 Department of Public Health, Yonsei University Graduate School, Seoul, South Korea; 2 Institute of Vision Research, Department of Ophthalmology, Yonsei University College of Medicine, Seoul, South Korea; 3 Department of Ophthalmology, Korea University College of Medicine, Seoul, South Korea; 4 Department of Preventive Medicine, Yonsei University College of Medicine, 50-1Yonsei-ro, Seodaemun-gu, Seoul 120-752, Republic of Korea

**Keywords:** Air pollutants, Dry eye disease, Meteorological factors, Prevalence, Spatial epidemiology

## Abstract

**Background:**

DED rate maps from diverse regions may allow us to understand world-wide spreading pattern of the disease. Only few studies compared the prevalence of DED between geographical regions in non-spatial context. Therefore, we examined the spatial epidemiological pattern of DED prevalence in South Korea using a nationally representative sample.

**Methods:**

We analyzed 16,431 Korean adults aged 30 years or older of the 5th Korea National Health and Nutrition Examination Survey. DED was defined as previously diagnosed by an ophthalmologist as well as symptoms experienced. Multiple logistic regression analysis was used to assess the spatial pattern in the prevalence of DED, and effects of environmental factors.

**Results:**

Among seven metropolitan cities and nine provinces, three metropolitan cities located in the southeast of Korea revealed the highest prevalence of DED. After adjusting for sex, age and survey year, people living in urban areas had higher risk of having DED. Adjusted odds ratio for having previously diagnosed DED was 1.677 (95% CI 1.299-2.166) for metropolitan cities and 1.580 (95% CI 1.215-2.055) for other cities compared to rural areas. Corresponding odds ratio for presenting DED symptoms was 1.388 (95% CI 1.090-1.766) for metropolitan cities and 1.271 (95% CI 0.999-1.617) for other cities. Lower humidity and longer sunshine duration were significantly associated with DED. Among air pollutants, SO_2_ was associated with DED, while NO_2_, O_3_, CO, and PM10 were not.

**Conclusion:**

Our findings suggest that prevalence of DED can be affected by the degree of urbanization and environmental factors such as humidity and sunshine duration.

## Background

Dry eye disease (DED) causes great discomfort on individual lives and is a rising public health issue. Previous studies revealed common symptoms of DED such as visual disturbance, ocular fatigue and pain are affecting the performance of daily activities and quality of life [1-7]. The prevalence of DED is continuously growing worldwide with a prevalence ranging from 4.3% to 73.5% [2-4,8-17], and it is one of the major reasons to seek eye care [6,7,18]. Studies indicated that old age and female sex are established risk factors of DED [3,10,14]. The prevalence of DED was comparably higher in Asian population than in Western population [9,11,12,17]. Yet, lack of effort in identifying the relationship between DED and region is largely unexplored. Within USA, prevalence of DED was the highest in south, but explanation on why south had the highest prevalence was not mentioned [12]. Another study performed in South Korea identified that DED prevalence was higher in urban areas than in rural areas [19]. However, this study was limited to the population of a single city, and regional characteristics were not investigated as main factors in relation to DED.

Examining geographical pattern in disease prevalence is important in epidemiology by providing health professionals with visual evidence for generating effective community-based strategies [20-22]. However, despite growing interest in this issue, only few studies empirically explored the spatial epidemiology of the prevalence of DED. Spatial epidemiology emerges as a key tool to identify the spread and possible causes of DED outbreaks since standard map display techniques enable the visualization of DED uncertainty and ensure more meaningful inferences from the spatial data. Inter-community DED figures compare the number of expected cases in the standard population with the number observed. It is expected that such direct standardization allows valid comparison of DED risks from exposure group in different countries.

Therefore, we investigated the spatial epidemiological pattern of DED prevalence in South Korea using the data from the 5th Korea National Health and Nutrition Examination Survey (KNHANES), and further assessed the effect of regional characteristics such as city size, meteorological factors and air pollutants. Although South Korea has been elected as a case study, this study may be relevant for other countries having similar spatial epidemiology contexts in relation to DED.

## Results

### Prevalence of DED by sex, age, and region

**Figure 1:**
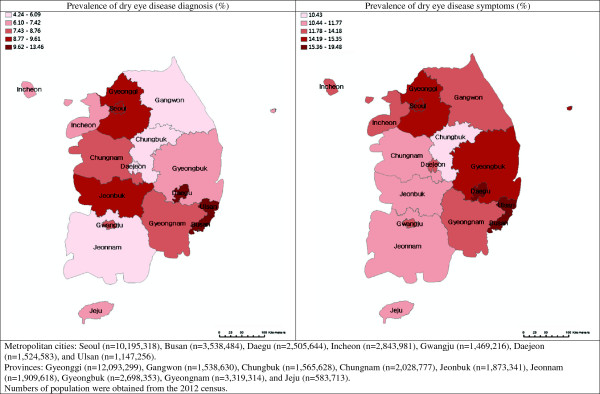
Prevalence of dry eye disease in metropolitan cities and provinces of South Korea.

Table 1 presents the prevalence of DED by sex, age, and region. Presence of DED was defined in two ways; having previously diagnosed DED and having DED symptoms. In this nationally representative sample, 10.4% (1,616 over 15,538) reported that they have been diagnosed as DED, and 17.7% (2,666 over 15,034) reported that they had symptoms of DED. A total of 1,314 people reported both previously diagnosed DED and DED symptoms. Both previously diagnosed DED and DED symptoms were more frequent in women (12.7% and 19.4%, respectively) than in men (4.6% and 9.8%, respectively). Prevalence of DED diagnosis and symptoms were higher in participants aged 60 to 69 (11.5% and 17.5%, respectively). Prevalence of DED diagnosis was highest in Ulsan (13.5%), followed by Busan (12.5%) and Daegu (10.6%). Similarly, DED symptoms were most frequent among participants residing in Busan (19.5%), Ulsan (16.8%), and Daegu (17.2%), though in a different order. Referring to Figure 1, which visually displays the prevalence of DED diagnosis and symptoms by region in South Korea, Ulsan, Busan, and Daegu metropolitan cities are located in the south east side of the peninsula.

**Table 1:** Prevalence of dry eye disease by participant’s characteristics

**Characteristics**		**No. of participants**	**Dry eye disease diagnosis**		**Dry eye disease symptoms**	
			**No.**	**Prevalence (95% CI)**	**No.**	**Prevalence (95% CI)**
Sex						
	Male	7033	374	4.60 (4.59-4.61)	745	9.84 (9.83-9.85)
	Female	9398	1242	12.65 (12.63-12.67)	1921	19.44 (19.42-19.46)
Age						
	30-39	3403	291	8.35 (8.33-8.37)	507	13.78 (13.76-13.80)
	40-49	3294	284	7.68 (7.66-7.70)	463	13.33 (13.31-13.35)
	50-59	3534	371	9.05 (9.03-9.07)	595	15.61 (15.58-15.64)
	60-69	3146	385	11.46 (11.43-11.49)	582	17.45 (17.41-17.49)
	70+	3054	285	8.21 (8.18-8.24)	519	15.40 (15.36-15.44)
Region						
	Seoul	3303	363	9.26 (9.24-9.28)	565	15.14 (15.11-15.17)
	Busan	985	137	12.52 (12.48-12.56)	189	19.48 (19.43-19.53)
	Daegu	817	94	10.56 (10.51-10.61)	140	17.23 (17.17-17.29)
	Incheon	934	76	6.84 (6.80-6.88)	139	13.98 (13.93-14.03)
	Gwangju	453	41	8.38 (8.32-8.44)	70	13.43 (13.36-13.50)
	Daejeon	499	38	6.09 (6.04-6.14)	69	13.22 (13.15-13.29)
	Ulsan	405	50	13.46 (13.38-13.54)	72	16.77 (16.69-16.85)
	Gyeonggi	3535	390	9.01 (8.99-9.03)	617	15.35 (15.32-15.38)
	Gangwon	527	38	5.30 (5.25-5.35)	84	13.28 (13.21-13.35)
	Chungbuk	517	43	5.57 (5.52-5.62)	70	10.43 (10.37-10.49)
	Chungnam	697	61	7.67 (7.63-7.71)	95	11.52 (11.47-11.57)
	Jeonbuk	685	65	9.61 (9.55-9.67)	85	11.43 (11.37-11.49)
	Jeonnam	707	34	4.24 (4.20-4.28)	92	11.77 (11.71-11.83)
	Gyeongbuk	1033	75	7.42 (7.38-7.46)	178	14.80 (14.75-14.85)
	Gyeongnam	969	92	8.76 (8.72-8.80)	148	14.18 (14.13-14.23)
	Jeju	365	19	6.50 (6.44-6.56)	53	11.63 (11.55-11.71)

Prevalence and confidence intervals were estimated with considering sampling weights.

Table 2 presents the results of serial logistic regression analyses. Compared to men, women had a higher odds ratio for previously diagnosed DED and symptoms of DED. People with age 60 to 69 had the highest odds ratio for DED diagnosis and DED symptoms. Prevalence of DED diagnosis showed statistically significant regional difference by revealing the highest odds in Ulsan (OR 1.74; 95% CI 1.19-2.54) and Busan (OR 1.47; 95% CI 1.04-2.06), and the lowest odds in Daejeon (OR 0.66; 95% CI 0.43-0.99), Gangwon (OR 0.55; 95% CI 0.36-0.84), and Jeonnam (OR 0.43; 95% CI 0.23-0.81). These associations remained significant after adjustment for sex, age, region, and survey year. Frequency of DED symptoms showed similar regional difference, though after adjustments the significance disappeared.

**Table 2:** Association between dry eye disease and participant’s characteristics

**Characteristics**		**Odds ratio (95% confidence interval) for dry eye disease diagnosis**			**Odds ratio (95% confidence interval) for dry eye disease symptoms**		
		**Unadjusted**	**Adjusted for sex and age**	**Adjusted for sex, age, region, and survey year**	**Unadjusted**	**Adjusted for sex and age**	**Adjusted for sex, age, region, and survey year**
Sex							
	Male	1.00	1.00	1.00	1.00	1.00	1.00
	Female	2.98 (2.58-3.45)	3.00 (2.59-3.46)	3.02 (2.61-3.50)	2.20 (1.95-2.47)	2.19 (1.95-2.46)	2.21 (1.96-2.48)
Age							
	30-39	1.00	1.00	1.00	1.00	1.00	1.00
	40-49	0.91 (0.74-1.13)	0.91 (0.73-1.13)	0.91 (0.73-1.13)	0.97 (0.81-1.16)	0.97 (0.80-1.16)	0.97 (0.80-1.16)
	50-59	1.07 (0.86-1.33)	1.06 (0.85-1.32)	1.06 (0.85-1.31)	1.12 (0.94-1.34)	1.12 (0.93-1.33)	1.11 (0.93-1.33)
	60-69	1.39 (1.13-1.71)	1.34 (1.09-1.66)	1.37 (1.11-1.68)	1.28 (1.08-1.53)	1.25 (1.05-1.49)	1.26 (1.06-1.51)
	70+	0.97 (0.77-1.22)	0.86 (0.68-1.08)	0.90 (0.71-1.14)	1.12 (0.93-1.35)	1.02 (0.84-1.23)	1.06 (0.87-1.29)
Region							
	Seoul	1.01 (0.81-1.26)	0.99 (0.79-1.23)	0.99 (0.79-1.24)	0.97 (0.78-1.22)	0.96 (0.76-1.20)	0.96 (0.76-1.20)
	Busan	1.47 (1.04-2.06)	1.42 (1.00-2.02)	1.43 (1.01-2.03)	1.41 (1.02-1.96)	1.38 (0.98-1.94)	1.37 (0.97-1.93)
	Daegu	1.19 (0.87-1.64)	1.15 (0.84-1.58)	1.16 (0.85-1.58)	1.35 (0.98-1.85)	1.29 (0.93-1.80)	1.28 (0.92-1.77)
	Incheon	0.72 (0.51-1.02)	0.71 (0.50-1.03)	0.72 (0.50-1.03)	0.88 (0.58-1.33)	0.87 (0.57-1.33)	0.87 (0.57-1.33)
	Gwangju	0.87 (0.49-1.56)	0.88 (0.48-1.61)	0.88 (0.51-1.51)	0.83 (0.47-1.47)	0.84 (0.47-1.47)	0.83 (0.49-1.41)
	Daejeon	0.66 (0.43-0.99)	0.64 (0.43-0.96)	0.61 (0.38-0.98)	0.82 (0.55-1.22)	0.81 (0.55-1.20)	0.78 (0.49-1.23)
	Ulsan	1.74 (1.19-2.54)	1.74 (1.17-2.59)	1.84 (1.25-2.71)	1.38 (0.79-2.41)	1.35 (0.77-2.36)	1.41 (0.86-2.33)
	Gyeonggi	1.00	1.00	1.00	1.00	1.00	1.00
	Gangwon	0.55 (0.36-0.84)	0.54 (0.35-0.83)	0.53 (0.34-0.84)	0.87 (0.55-1.35)	0.85 (0.54-1.33)	0.84 (0.55-1.29)
	Chungbuk	0.58 (0.30-1.10)	0.59 (0.32-1.11)	0.62 (0.33-1.18)	0.61 (0.26-1.43)	0.62 (0.27-1.43)	0.65 (0.29-1.44)
	Chungnam	0.81 (0.48-1.36)	0.78 (0.46-1.30)	0.78 (0.47-1.30)	0.72 (0.43-1.20)	0.68 (0.41-1.14)	0.68 (0.41-1.13)
	Jeonbuk	1.04 (0.69-1.58)	1.01 (0.66-1.54)	1.01 (0.67-1.52)	0.70 (0.47-1.07)	0.68 (0.45-1.04)	0.68 (0.45-1.03)
	Jeonnam	0.43 (0.23-0.81)	0.39 (0.21-0.74)	0.39 (0.20-0.74)	0.73 (0.45-1.20)	0.67 (0.40-1.11)	0.65 (0.39-1.09)
	Gyeongbuk	0.81 (0.50-1.30)	0.77 (0.47-1.26)	0.78 (0.48-1.27)	0.97 (0.67-1.40)	0.93 (0.64-1.36)	0.94 (0.64-1.37)
	Gyeongnam	0.94 (0.66-1.34)	0.93 (0.65-1.32)	0.92 (0.65-1.31)	0.92 (0.66-1.27)	0.90 (0.65-1.24)	0.89 (0.65-1.22)
	Jeju	0.68 (0.46-1.00)	0.67 (0.45-1.00)	0.71 (0.45-1.10)	0.83 (0.51-1.35)	0.79 (0.46-1.36)	0.83 (0.46-1.52)

Odds ratio and confidence intervals were estimated with considering sampling weights.

### Relationship between DED and regional characteristics

Table 3 presents the relationship between regional characteristics and the prevalence of DED. In the unadjusted analysis, both diagnosis and symptoms of DED revealed significantly higher odds in metropolitan cities compared to rural areas with an OR of 1.654 (95% CI 1.297-2.110) and 1.352 (95% CI 1.075-1.700), respectively. After adjustment, the associations were still consistently significant. Non-metropolitan cities also revealed higher odds for DED than rural areas. However, the difference between non-metropolitan cities and rural area was statistically significant for DED diagnosis, but not for symptoms of DED. Moving on to meteorological factors**,** prevalence of previous DED diagnosis was positively associated with higher temperature (OR 1.076; 95% CI 1.009-1.148) and longer sunshine duration (OR 1.015; 95% CI 1.006-1.023), and negatively associated with higher humidity (OR 0.970; 95% CI 0.955-0.986) after adjusting for sex, age, and survey year. Presenting DED symptoms was positively associated with longer sunshine duration (OR 1.013; 95% CI 1.005-1.021) and negatively associated with higher humidity (OR 0.975; 95% CI 0.959-0.991) when adjusted for sex, age, and survey year. Among the five air pollutants analyzed, only SO_2_ concentration had a significantly positive association with DED diagnosis (OR 1.092; 95% CI 1.011-1.179) and DED symptoms (OR 1.092; 95% CI 1.005-1.187) after adjusting for sex, age, and survey year.

**Table 3:** Association between dry eye disease and regional characteristics

**Regional characteristics**		**Odds ratio (95% confidence interval) for dry eye disease diagnosis**			**Odds ratio (95% confidence interval) for dry eye disease symptoms**		
		**Unadjusted**	**Adjusted for sex and age**	**Adjusted for sex, age, and survey year**	**Unadjusted**	**Adjusted for sex and age**	**Adjusted for sex, age, and survey year**
City Size							
	Metropolitan Cities	1.654 (1.297-2.110)	1.689 (1.314-2.171)	1.677 (1.299-2.166)	1.352 (1.075-1.700)	1.390 (1.096-1.765)	1.388 (1.090-1.766)
	Other Cities	1.554 (1.206-2.002)	1.594 (1.229-2.067)	1.580 (1.215-2.055)	1.229 (0.970-1.558)	1.272 (0.996-1.624)	1.271 (0.999-1.617)
	Rural	1.00	1.00	1.00	1.00	1.00	1.00
Meteorological factor (1-SD increase)							
	Average Temperature	1.073 (1.009-1.141)	1.069 (1.004-1.138)	1.076 (1.009-1.148)	1.073 (1.007-1.145)	1.067 (0.998-1.140)	1.065 (0.996-1.138)
	Average Wind Speed	1.028 (0.912-1.158)	1.019 (0.902-1.151)	1.009 (0.892-1.140)	1.075 (0.952-1.214)	1.064 (0.939-1.205)	1.054 (0.928-1.198)
	Average Humidity	0.964 (0.949-0.979)	0.964 (0.949-0.979)	0.970 (0.955-0.986)	0.971 (0.956-0.987)	0.971 (0.956-0.987)	0.975 (0.959-0.991)
	Sunshine Duration	1.016 (1.010-1.021)	1.016 (1.010-1.022)	1.015 (1.006-1.023)	1.012 (1.008-1.017)	1.013 (1.008-1.018)	1.013 (1.005-1.021)
	Precipitation	1.000 (0.997-1.003)	1.000 (0.997-1.003)	1.002 (0.999-1.006)	0.998 (0.995-1.000)	0.998 (0.995-1.000)	1.000 (0.997-1.003)
Air pollutant (1-SD increase)							
	Sulfur dioxide	1.083 (1.007-1.165)	1.082 (1.003-1.168)	1.092 (1.011-1.179)	1.087 (1.001-1.181)	1.087 (1.000-1.182)	1.092 (1.005-1.187)
	Nitrogen dioxide	1.006 (0.994-1.018)	1.007 (0.994-1.019)	1.011 (0.998-1.024)	1.003 (0.991-1.015)	1.005 (0.993-1.017)	1.009 (0.996-1.022)
	Ozone	1.010 (0.989-1.031)	1.009 (0.987-1.031)	0.991 (0.968-1.014)	1.011 (0.992-1.031)	1.009 (0.988-1.030)	0.995 (0.971-1.019)
	Carbon monoxide	0.999 (0.998-1.000)	0.999 (0.998-1.000)	0.999 (0.998-1.000)	0.999 (0.998-1.001)	1.000 (0.998-1.001)	1.000 (0.999-1.001)
	PM10	0.987 (0.974-1.000)	0.988 (0.974-1.001)	1.014 (0.998-1.031)	0.987 (0.974-1.000)	0.988 (0.974-1.001)	1.010 (0.992-1.027)

Odds ratio and confidence intervals were estimated with considering sampling weights.

Post-hoc subgroup analyses were performed by survey year and participant’s sex. Urban areas revealed a higher prevalence of DED compared to rural areas regardless of survey year, though the overall prevalence increased between 2010 and 2012 (Additional file 1: Tables S1 and S2). Similar regional patterns of DED prevalence were observed for men and women (Additional file 1: Tables S3 and S4).

## Discussion

South Korea is located in East Asia with large numbers of mountains and rivers crisscrossing the peninsula. The country is divided into notable urban and rural areas with four distinct seasons. The population is a relatively homogeneous society with an absolute majority of Korean ethnicity [19]. Even though South Korea’s land area is small, due to its unique characteristics this nation is a suitable site to compare the prevalence of DED between urban and rural areas [19]. We did not investigate the effects of ethnicity on DED, but the ethnicity might not contribute to the prevalence of DED in the present study as reported in the West.

Similar to previous studies [2,3,8,12,14], our findings also reported that middle aged or older women has an increased risk of suffering from DED. Our study also observed that people in their age 60s reported highest frequency of previous DED diagnosis as well as symptoms of DED. Moving on to region, our results pointed out that the prevalence of DED is higher in most metropolitan cities. Ulsan metropolitan city marked the highest prevalence and the highest odds for previously diagnosed DED before and after sex, age, region, and survey year adjustment. Ulsan is the most industrialized city and takes the role of maintaining and advancing the Korean economy. According to a study evaluating air pollution and mortality in Seoul and Ulsan metropolitan cities, industrial sources were the major contributing factor of air pollution in Ulsan [23]. This reason might have contributed at least partially to the high prevalence of DED in Ulsan. Busan metropolitan city marked the second highest prevalence and significantly higher odds for DED diagnosis. Busan has the second biggest population and the largest container handling port in South Korea. Daegu showed the third highest prevalence of DED among all regions. Daegu is the fourth largest city in South Korea and has a valley form of terrain, because it is surrounded by low mountains. Since it is trapped by mountains, among 16 administrative districts, Daegu shows the highest temperature in summer in South Korea.

Similar to our results, a few studies observed regional difference of DED, nevertheless none of the studies used spatial epidemiological pattern as a major independent variable [3,19,24]. According to a study in Taiwan, majority of patients with DED were residing in urbanized areas [24]. Another study in mainland China also showed that DED symptoms were significantly associated with urban regions [3]. Two studies in Korea showed comparably similar results as our results, though in a non-spatial context. One study compared the prevalence of DED between urban and rural areas within Yongin city, which is located in the Gyeonggi province of South Korea. As a result, residents with DED were mostly residing in urban areas within Yongin city [19]. However, Yongin city is too small to represent the characteristics of urban and rural residents of Korea. Another study analyzed the data collected from KNHANES 2010–2012 and observed that the prevalence of DED among women was low in rural areas [25]. A study in Koumi town, Japan, a rural mountain area, reported the prevalence of clinically diagnosed DED and severe symptoms of DED in men as 2.1% and 11.5%, respectively, and 7.9% and 18.7% in women. After combining the percentages for both clinically diagnosed and severe symptoms of DED, 12.5% and 21.6% of men and women were suffering from DED, respectively [26]. The combined prevalence were higher than the prevalence reported from the West [12], but still lower than studies from the East [2,9] which mainly targeted urban areas, and exclusively, the prevalence for clinically diagnosed DED was significantly low compared to the East and West urban regions. Unlike these findings, a study held in Rajasthan, India reported that the prevalence of DED was higher in rural residents (19.6%) compared to urban dwellers (17.5%) [27], but the association was not statistically significant. This cross-sectional study [27] randomly selected 500 hospital-based patients who presented various ophthalmic problems to a tertiary eye care center and were screened for DED. Rajasthan has a population of 70 million people, and its land area is three times larger than the entire land of South Korea. The hospital-based sample of 500 patients might not represent the DED patients in the total population of Rajasthan. The authors suggested that overwhelming exposure to sunlight, high temperature, and excessive wind contributes to the higher prevalence of DED among rural residents [27].

Spatial pattern of DED might be, at least partially, due to difference of environmental and lifestyle factors between urban and rural areas, though lifestyle factors have not been analyzed in this paper. Regarding meteorological conditions, previous studies suggested humidity, wind speed, and sunshine duration as potential risk factors of DED [27-32]. Our results showed that lower humidity and longer sunshine duration were associated with DED before and after adjusting for covariates. A previous study developed a controlled-environment chamber (CEC) to verify the effects of a low humidity setting on ocular surface in normal mice and found that CEC-kept mice had decreased tear secretion and increased corneal fluorescein staining compared to control mice [30]. Human studies also observed that tear evaporative rate, tear volume, epidermal growth factor, and blink rate were associated with humid condition [28,29,31,32]. Relatively low humidity may increase tear evaporative rate not only in DED patients but also in healthy subjects [28,29,31,32]. Air pollution can be another potential risk factor of DED [27,33]. In our study, SO_2_ concentration was the only factor associated with DED after adjusted for sex, age and survey year. Concentration of CO was associated with DED when unadjusted or sex- and age- adjusted. Though further investigation is required for the association between DED and air pollutants, our results indicate that chronic exposure to disturbing environment can be a potential risk factor of DED.

Our study has several limitations to be discussed. First, this study used questionnaire-based assessment to estimate prevalence of DED. Nevertheless, questionnaire interview on DED diagnosis and symptoms were done by trained ophthalmologists. Evaluation of eye disease in the KNHANES was performed and monitored by the Korean Ophthalmological Society. According to a survey by the Korean Corneal Disease Study Group, 79% of corneal subspecialists use the Dry Eye Workshop classification [18] and 21% use the Dysfunctional Tear Syndrome Study Group guidelines [34] to diagnose DED [35]. This states that most ophthalmologists follows a certain guideline when diagnosing DED, however not knowing which diagnostic criteria used is one of the limitations for this study. The KNHANES questionnaire for DED symptoms included only dryness and irritation, therefore lack of information on detecting DED symptoms may lead to misclassification. However, studies reported significant associations between clinical signs and symptoms of DED [36-38]. Subjective method of DED diagnosis also has a clinical value, since DED symptoms directly affect the quality of life [35,39]. Second, the KNHANES was designed to estimate disease prevalence for the entire Korean population and for each metropolitan city or province. Thus, we were unable to investigate the effects caused by regional characteristics at smaller area units. Third, we did not controlled socioeconomic factors, medical history, and other variables that might be linked with DED. Lastly, the analysis involved residents of only in Korea, and therefore the results may not be generalizable to other populations. Despite these limitations, this is the first study that compared the prevalence of DED in different regions of South Korea, by using visual aid and statistical analysis along with environmental factors. This study used a large nationally representative dataset with a stratified analysis with sufficient statistical power.

## Conclusions

In conclusion, the prevalence of DED differs by region revealing a high prevalence and significant association in most metropolitan cities. Meteorological factors such as annual average humidity and sunshine duration were associated with DED. Further investigation is required to determine whether specific air pollutants play a role in the development of DED. Additionally, more studies are needed to establish the causal effects of environmental drivers such as urbanization, meteorological factors, air pollutants, and population density on the development of DED.

This paper contributes to the literature by presenting comparable spatial epidemiological pattern of the prevalence of DED. The large nationwide sample allowed us to examine whether the prevalence of DED varied according to the urban, rural, and industrial areas. Although further research is required, the results confirm the regional difference of DED prevalence and provide a basis for comparison with other countries and a methodological framework for examining spatial epidemiological pattern of DED in the context of worldwide regional communities.

## Methods

### Study subjects

Data for this study were derived from the 5th Korea National Health and Nutrition Examination Survey (KNHANES) which was conducted from 2010 to 2012 by the Korea Centers for Disease Control and Prevention (KCDC). Between 1998 and 2005, the KNHANES has been performed every three years as a short-term research operating system, but the survey has been conducted annually since 2007. Survey data from 2007 to 2009 were collected and named as the 4th KNHANES and survey data for the 5th KNHANES were collected during 2010 through 2012. Among 16,431 participants aged 30 years or older, 893 people were non-responsive for previous DED diagnosis, and 1,397 were non-responsive for DED symptoms. Thus, 15,538 and 15,034 participants were analyzed for previous DED diagnosis and DED symptoms, respectively. Each year, the survey protocol and informed consent forms are approved by the Institutional Review Board of the KCDC. The KNHANES complied with the tenets of the Declaration of Helsinki. The KNHANES is an ongoing nationally representative study, and de-identified datasets are publicly available from the KCDC. More detailed description of the KNHANES can be found elsewhere [40,41].

Regarding sampling units, the KNHANES uses a stratified multistage probability sampling design based on geographic area, sex, and age group to obtain information that represents non-institutionalized civilian population of South Korea. The survey consists of three parts: health interview survey, health examination survey, and nutrition survey. Survey questionnaires were asked by a trained staff, and the examination components were administered by highly trained medical personnel. Demographic information of each participant was collected from a prepared questionnaire. Ophthalmologic examinations were included first in 2008, and questionnaires on DED were introduced since 2010. South Korea is divided into 16 administrative divisions including nine provinces (Gyeonggi, Gangwon, Chungbuk, Chungnam, Jeonbuk, Jeonnam, Gyeongbuk, Gyeongnam, and Jeju), six metropolitan cities (Busan, Daegu, Incheon, Gwangju, Daejeon, and Ulsan), and one capital metropolitan city (Seoul). Korea’s administrative regions can be classified into three groups based on the population size: counties (less than 50,000), cities (more than 50,000), and metropolitan cities (more than 1,000,000). The six provinces are composed of over 200 counties and cities. Non-metropolitan cities within a province have a characteristic of an urbanized city, and counties resemble the nature of a typical rural region. To analyze the representative national data of the prevalence of DED in South Korea, three years’ datasets (2010, 2011 and 2012) were combined.

### Dry eye disease

Ophthalmologists designated by the Korean Ophthalmological Society performed eye examinations. The KCDC and the Korean Ophthalmological Society conducted education and training program twice a year. Educational information included the overall purpose of eye disease epidemiological studies, cautions, machine operation, and diagnosis and classification of major eye disorders. Eye disease questionnaires included frequency of eye screening, hours of sun exposure, family history of eye disease, past history of eye disease including DED, glaucoma, cataracts, and age-related macular degeneration.

### Environmental factors

Meteorological factors for each administrative district since 2010 to 2012 were obtained from the National Climate Data Service System. Average temperature and humidity were measured 8 times 3 hours apart for every single day. Average wind speed was divided by the total value of artistic air for the entire day over 86,400 seconds. Sunshine duration was defined as hours of sun rays without blockage of clouds or fogs. Precipitation was measured by the total amount of precipitation for 24 hours, daily. Data for regional air pollutants were acquired from the Ministry of Environment. SO_2_, NO_2_, O_3_, CO, and PM10 were measured and analyzed at 250 national metrological locations in South Korea. For our analysis annual average values were encountered.

### Statistical analysis

Weighted analyses were performed for prevalence and odds ratio of DED, since KNHANES provides weights to compensate with its complex sampling design [25]. Serial multiple logistic regression models were used to calculate the odds ratio (OR) and 95% confidence interval (95% CI) for previously diagnosed DED and symptoms. Gyeonggi province was selected as a reference for analyzing the association between DED and region, because Gyeonggi had the highest number of people who participated in the survey. The above analyzing method was used once again to determine the association between DED and environmental factors (test of odds ratio for a 1-SD increase). Statistical analyses were performed using SAS Version 9.2 (SAS Institute, Inc. Cary, NC). To visually display the prevalence of DED by region, ArcGIS Version 10.1 (Esri Headquarters, Redlands, CA) was used.

## Abbreviations

CI: Confidence interval; DED: Dry eye disease; KCDC: Korea center for disease control and prevention; KNHANES: Korea national health and nutrition examination survey; OR: Odds ratio; SD: Standard deviation.

## Competing interests

The authors declare that they have no competing interest.

## Author’s contributions

SBU searched literatures, analyzed data and wrote the manuscript. NHK assisted with the statistical analyses. HKL and JSS critically revised and helped to draft the manuscript. HCK conceptualized the entire manuscript by providing guidance in designing analyses and contributed to writing the manuscript. All authors edited and approved the final manuscript.

## Supplementary Material

Additional file 1: Table S1 Change in previous dry eye disease diagnosis between 2010 and 2012 by region. **Table S2.** Change in dry eye disease symptoms experienced between 2010 and 2012 by region. **Table S3.** Prevalence of and odds ratio for dry eye disease in men. **Table S4.** Prevalence of and odds ratio for dry eye disease in women. Click here for file
