# Supplementary material for: Spatial epidemiology of dry eye disease: findings from South Korea
Source: Int J Health Geogr. 2014 Aug 15;13:31. doi: 10.1186/1476-072X-13-31 (PMC4139141; doi:10.1186/1476-072X-13-31)
Supplement: Additional file 1: Table S1 — Change in previous dry eye disease diagnosis between 2010 and 2012 by region. Table S2. Change in dry eye disease symptoms experienced between 2010 and 2012 by region. Table S3. Prevalence of and odds ratio for dry eye disease in men. Table S4. Prevalence of and odds ratio for dry eye disease in women. [file 1476-072X-13-31-S1.docx]

| Additional file 1: Table S1. Change in previous dry eye disease diagnosis between 2010 and 2012 by region | | | | | |
| --- | --- | --- | --- | --- | --- |
| City Size | 2010 | |  | 2012 | |
|  | Prevalence (95% CI) | Sex and age adjusted OR (95% CI) |  | Prevalence (95% CI) | Sex and age adjusted OR (95% CI) |
| Seoul Metropolitan City | 9.10 (9.08-9.16) | 1.94 (1.23-3.08) |  | 11.02 (11.00-11.04) | 1.87 (1.17-2.99) |
| Metropolitan Cities | 7.16 (7.14-7.18) | 1.47 (0.94-2.30) |  | 13.11 (13.09-13.13) | 2.43 (1.51-3.92) |
| Other Cities | 7.89 (7.87-7.91) | 1.66 (1.08-2.56) |  | 12.53 (12.51-12.55) | 2.19 (1.40-3.41) |
| Rural | 5.32 (5.30-5.34) | 1.00 |  | 6.45 (6.43-6.47) | 1.00 |
| Metropolitan Cities | 8.04 (8.03-8.05) | 1.68 (1.11-2.53) |  | 12.21 (12.19-12.23) | 2.18 (1.40-3.38) |
| Other Cities | 7.89 (7.87-7.91) | 1.66 (1.08-2.56) |  | 12.53 (12.51-12.55) | 2.19 (1.40-3.41) |
| Rural | 5.32 (5.30-5.34) | 1.00 |  | 6.45 (6.43-6.47) | 1.00 |
| Urban | 7.92 (7.91-7.93) | 1.56 (1.07-2.26) |  | 12.34 (12.33-12.35) | 2.16 (1.42-3.27) |
| Rural | 5.65 (5.63-5.67) | 1.00 |  | 6.51 (6.49-6.53) | 1.00 |
| Prevalence (95% CI) and odds ratio (95% CI) were estimated with considering sampling weights | | | | | |

| Additional file 1: Table S2. Change in dry eye disease symptoms experienced between 2010 and 2012 by region | | | | | |
| --- | --- | --- | --- | --- | --- |
| City Size | 2010 | |  | 2012 | |
|  | Prevalence (95% CI) | Sex and age adjusted OR (95% CI) |  | Prevalence (95% CI) | Sex and age adjusted OR (95% CI) |
| Seoul Metropolitan City | 13.82 (13.79-13.85) | 1.33 (0.83-2.12) |  | 16.62 (16.59-16.65) | 1.24 (0.83-1.86) |
| Metropolitan Cities | 17.42 (17.39-17.45) | 1.74 (1.08-2.82) |  | 19.39 (19.36-19.42) | 1.60 (1.12-2.29) |
| Other Cities | 12.39 (12.37-12.41) | 1.17 (0.76-1.80) |  | 21.87 (21.85-21.89) | 1.77 (1.27-2.47) |
| Rural | 11.68 (11.66-11.70) | 1.00 |  | 14.01 (13.98-14.04) | 1.00 |
| Metropolitan Cities | 15.78 (15.76-15.80) | 1.55 (1.01-2.38) |  | 18.19 (18.17-18.21) | 1.44 (1.03-2.01) |
| Other Cities | 12.39 (12.37-12.41) | 1.17 (0.76-1.80) |  | 21.87 (21.85-21.89) | 1.77 (1.27-2.47) |
| Rural | 11.68 (11.66-11.70) | 1.00 |  | 14.01 (13.98-14.04) | 1.00 |
| Urban | 14.38 (14.37-14.39) | 1.37 (0.94-2.02) |  | 19.75 (19.73-19.77) | 1.58 (1.16-2.16) |
| Rural | 11.79 (11.77-11.81) | 1.00 |  | 13.96 (13.93-13.99) | 1.00 |
| Prevalence (95% CI) and odds ratio (95% CI) were estimated with considering sampling weights | | | | | |

| Additional file 1: Table S3. Prevalence of and odds ratio for dry eye disease in men | | | | | | | | | | | |
| --- | --- | --- | --- | --- | --- | --- | --- | --- | --- | --- | --- |
| Characteristics | | No. of participants | Dry eye disease diagnosis | | | |  | Dry eye disease symptoms | | | |
|  |  |  | No. | Prevalence  (95% CI) | Unadjusted OR (95% CI) | Age, survey year and region adjusted  OR (95% CI) |  | No. | Prevalence  (95% CI) | Unadjusted  OR (95% CI) | Age, survey year and region adjusted  OR (95% CI) |
| Age |  |  |  |  |  |  |  |  |  |  |  |
|  | 30-39 | 1303 | 57 | 4.68 (4.66-4.70) | 1.00 | 1.00 |  | 131 | 9.94 (9.91-9.97) | 1.00 | 1.00 |
|  | 40-49 | 1340 | 66 | 4.49 (4.47-4.51) | 0.96 (0.62-1.49) | 0.98 (0.63-1.52) |  | 140 | 10.00 (9.97-10.03) | 1.00 (0.73-1.38) | 1.01 (0.74-1.39) |
|  | 50-59 | 1406 | 63 | 3.53 (3.51-3.55) | 0.75 (0.48-1.16) | 0.75 (0.49-1.17) |  | 136 | 9.73 (9.70-9.76) | 0.97 (0.71-1.32) | 0.98 (0.71-1.34) |
|  | 60-69 | 1343 | 108 | 8.14 (8.10-8.18) | 1.81 (1.23-2.65) | 1.87 (1.28-2.75) |  | 176 | 12.33 (12.28-12.38) | 1.27 (0.93-1.72) | 1.30 (0.95-1.77) |
|  | 70+ | 1222 | 80 | 5.60 (5.56-5.64) | 1.21 (0.81-1.81) | 1.29 (0.86-1.94) |  | 162 | 12.43 (12.38-12.49) | 1.27 (0.93-1.73) | 1.34 (0.97-1.83) |
| Region |  |  |  |  |  |  |  |  |  |  |  |
|  | Seoul | 1347 | 97 | 5.47 (5.44-5.50) | 1.02 (0.71-1.46) | 1.00 (0.70-1.44) |  | 162 | 10.16 (10.13-10.19) | 0.84 (0.60-1.16) | 0.83 (0.60-1.16) |
|  | Busan | 367 | 20 | 4.24 (4.20-4.28) | 0.78 (0.42-1.46) | 0.75 (0.40-1.42) |  | 44 | 13.73 (13.66-13.80) | 1.21 (0.69-2.14) | 1.18 (0.66-2.13) |
|  | Daegu | 322 | 17 | 4.47 (4.42-4.52) | 0.82 (0.40-1.69) | 0.82 (0.40-1.67) |  | 42 | 12.28 (12.20-12.36) | 1.23 (0.73-2.06) | 1.18 (0.71-1.98) |
|  | Incheon | 374 | 15 | 3.86 (3.82-3.90) | 0.71 (0.34-1.45) | 0.71 (0.34-1.46) |  | 38 | 9.71 (9.65-9.77) | 0.79 (0.48-1.30) | 0.79 (0.48-1.29) |
|  | Gwangju | 196 | 12 | 5.80 (5.73-5.87) | 1.08 (0.48-2.46) | 1.09 (0.49-2.39) |  | 21 | 10.34 (10.25-10.43) | 0.89 (0.49-1.61) | 0.89 (0.48-1.63) |
|  | Daejeon | 214 | 7 | 1.17 (1.14-1.20) | 0.21 (0.08-0.53) | 0.19 (0.07-0.53) |  | 16 | 7.49 (7.41-7.57) | 0.58 (0.29-1.71) | 0.55 (0.26-1.15) |
|  | Ulsan | 140 | 12 | 11.66 (11.55-11.77) | 2.33 (1.27-4.27) | 2.51 (1.39-4.50) |  | 20 | 15.70 (15.58-15.83) | 1.59 (0.87-2.92) | 1.6 (0.92-3.00) |
|  | Gyeonggi | 1398 | 93 | 5.37 (5.35-5.39) | 1.00 | 1.00 |  | 184 | 12.05 (12.02-12.08) | 1.00 | 1.00 |
|  | Gangwon | 225 | 13 | 5.48 (5.41-5.55) | 1.02 (0.53-1.98) | 1.01 (0.50-2.03) |  | 28 | 10.62 (10.53-10.71) | 0.93 (0.51-1.68) | 0.92 (0.50-1.66) |
|  | Chungbuk | 228 | 13 | 2.92 (2.87-2.97) | 0.53 (0.26-1.10) | 0.56 (0.26-1.20) |  | 22 | 5.99 (5.92-6.06) | 0.47 (0.13-1.62) | 0.49 (0.14-1.63) |
|  | Chungnam | 291 | 12 | 4.33 (4.28-4.38) | 0.80 (0.36-1.76) | 0.75 (0.34-1.62) |  | 19 | 5.88 (5.82-5.94) | 0.49 (0.22-1.11) | 0.46 (0.21-1.04) |
|  | Jeonbuk | 289 | 13 | 4.53 (4.47-4.59) | 0.84 (0.41-1.69) | 0.78 (0.38-1.60) |  | 23 | 7.18 (7.11-7.25) | 0.57 (0.31-1.05) | 0.54 (0.29-1.01) |
|  | Jeonnam | 292 | 5 | 1.41 (1.38-1.44) | 0.25 (0.08-0.79) | 0.23 (0.07-0.72) |  | 26 | 8.21 (8.13-8.29) | 0.70 (0.38-1.31) | 0.64 (0.34-1.23) |
|  | Gyeongbuk | 407 | 22 | 6.25 (6.20-6.30) | 1.18 (0.61-2.25) | 1.13 (0.59-2.17) |  | 54 | 11.83 (11.76-11.90) | 1.00 (0.58-1.70) | 0.96 (0.56-1.67) |
|  | Gyeongnam | 377 | 17 | 4.19 (4.15-4.23) | 0.77 (0.35-1.68) | 0.76 (0.35-1.65) |  | 30 | 9.41 (9.37-9.45) | 0.76 (0.45-1.27) | 0.74 (0.44-1.24) |
|  | Jeju | 147 | 6 | 4.27 (4.20-4.34) | 0.79 (0.31-2.01) | 0.83 (0.30-2.25) |  | 16 | 6.45 (6.36-6.54) | 0.63 (0.19-2.15) | 0.67 (0.17-2.52) |
| Prevalence (95% CI) and odds ratio (95% CI) were estimated with considering sampling weights | | | | | | | | | | | |

| Additional file 1: Table S4. Prevalence of and odds ratio for dry eye disease in women | | | | | | | | | | | |
| --- | --- | --- | --- | --- | --- | --- | --- | --- | --- | --- | --- |
| Characteristics | | No. of participants | Dry eye disease diagnosis | | | |  | Dry eye disease symptoms | | | |
|  |  |  | No. | Prevalence  (95% CI) | Unadjusted OR (95% CI) | Age, survey year and region adjusted  OR (95% CI) |  | No. | Prevalence  (95% CI) | Unadjusted  OR (95% CI) | Age, survey year and region adjusted  OR (95% CI) |
| Age |  |  |  |  |  |  |  |  |  |  |  |
|  | 30-39 | 1875 | 234 | 13.31 (13.28-13.34) | 1.00 | 1.00 |  | 376 | 19.66 (19.62-19.70) | 1.00 | 1.00 |
|  | 40-49 | 1738 | 218 | 11.97 (11.94-12.00) | 0.89 (0.70-1.13) | 0.89 (0.70-1.13) |  | 323 | 18.53 (18.49-18.57) | 0.94 (0.77-1.15) | 0.94 (0.77-1.15) |
|  | 50-59 | 1965 | 308 | 15.35 (15.31-15.39) | 1.18 (0.92-1.52) | 1.18 (0.92-1.52) |  | 459 | 22.87 (22.83-22.91) | 1.20 (0.98-1.47) | 1.19 (0.97-1.46) |
|  | 60-69 | 1674 | 277 | 15.22 (15.28-15.38) | 1.18 (0.93-1.50) | 1.20 (0.94-1.53) |  | 406 | 23.42 (23.36-23.48) | 1.24 (1.02-1.52) | 1.25 (1.02-1.53) |
|  | 70+ | 1672 | 205 | 10.58 (10.54-10.62) | 0.77 (0.59-1.01) | 0.81 (0.62-1.07) |  | 357 | 18.65 (18.60-18.70) | 0.93 (0.75-1.16) | 0.96 (0.77-1.20) |
| Region |  |  |  |  |  |  |  |  |  |  |  |
|  | Seoul | 1789 | 266 | 13.75 (13.71-13.79) | 0.99 (0.77-1.29) | 0.98 (0.75-1.28) |  | 403 | 21.32 (21.27-21.37) | 1.05 (0.82-1.34) | 1.04 (0.81-1.33) |
|  | Busan | 544 | 117 | 21.96 (21.88-22.04) | 1.76 (1.24-2.49) | 1.74 (1.20-2.48) |  | 145 | 27.66 (27.58-27.74) | 1.52 (1.07-2.18) | 1.49 (1.05-2.13) |
|  | Daegu | 447 | 77 | 17.26 (17.18-17.34) | 1.30 (0.90-1.88) | 1.30 (0.89-1.88) |  | 98 | 23.77 (23.68-23.86) | 1.40 (0.96-1.96) | 1.34 (0.93-1.93) |
|  | Incheon | 524 | 61 | 10.34 (10.27-10.41) | 0.72 (0.47-1.10) | 0.72 (0.47-1.10) |  | 101 | 19.37 (19.28-19.46) | 0.93 (0.59-1.48) | 0.93 (0.59-1.47) |
|  | Gwangju | 251 | 29 | 11.24 (11.14-11.34) | 0.79 (0.42-1.47) | 0.79 (0.45-1.40) |  | 49 | 16.97 (16.86-17.09) | 0.79 (0.40-1.57) | 0.80 (0.43-1.50) |
|  | Daejeon | 261 | 31 | 11.44 (11.35-11.53) | 0.81 (0.52-1.26) | 0.77 (0.47-1.26) |  | 53 | 20.28 (20.16-20.40) | 0.95 (0.61-1.49) | 0.91 (0.55-1.53) |
|  | Ulsan | 213 | 38 | 19.27 (19.14-19.40) | 1.49 (0.96-2.32) | 1.57 (1.01-2.44) |  | 52 | 22.91 (22.77-23.05) | 1.22 (0.60-2.49) | 1.29 (0.65-2.55) |
|  | Gyeonggi | 1916 | 297 | 13.82 (13.78-13.86) | 1.00 | 1.00 |  | 433 | 20.69 (20.65-20.73) | 1.00 | 1.00 |
|  | Gangwon | 283 | 25 | 5.61 (5.54-5.68) | 0.37 (0.16-0.85) | 0.37 (0.15-0.87) |  | 56 | 17.05 (16.94-17.16) | 0.82 (0.50-1.33) | 0.80 (0.51-1.26) |
|  | Chungbuk | 270 | 30 | 9.13 (9.04-9.22) | 0.63 (0.32-1.25) | 0.65 (0.33-1.30) |  | 48 | 16.48 (16.37-16.60) | 0.74 (0.35-1.56) | 0.76 (0.38-1.55) |
|  | Chungnam | 387 | 49 | 11.15 (11.08-11.22) | 0.78 (0.46-1.33) | 0.79 (0.47-1.34) |  | 76 | 17.31 (17.22-17.40) | 0.82 (0.48-1.40) | 0.82 (0.48-1.39) |
|  | Jeonbuk | 375 | 52 | 15.14 (15.04-15.24) | 1.11 (0.70-1.76) | 1.12 (0.71-1.74) |  | 62 | 16.32 (16.22-16.42) | 0.78 (0.47-1.31) | 0.77 (0.47-1.27) |
|  | Jeonnam | 399 | 29 | 6.76 (6.70-6.82) | 0.45 (0.24-0.85) | 0.44 (0.23-0.84) |  | 66 | 15.30 (15.21-15.39) | 0.70 (0.40-1.20) | 0.66 (0.38-1.14) |
|  | Gyeongbuk | 540 | 53 | 9.37 (9.31-9.43) | 0.65 (0.37-1.12) | 0.65 (0.37-1.15) |  | 124 | 19.27 (19.19-19.35) | 0.94 (0.65-1.35) | 0.93 (0.64-1.34) |
|  | Gyeongnam | 548 | 75 | 13.85 (13.78-13.92) | 1.00 (0.71-1.41) | 1.00 (0.71-1.40) |  | 118 | 20.24 (20.16-20.32) | 1.00 (0.71-1.42) | 0.98 (0.70-1.39) |
|  | Jeju | 177 | 13 | 9.12 (9.02-9.22) | 0.63 (0.43-0.92) | 0.66 (0.44-0.99) |  | 37 | 17.47 (17.34-17.61) | 0.90 (0.65-1.26) | 0.94 (0.65-1.36) |
| Prevalence (95% CI) and odds ratio (95% CI) were estimated with considering sampling weights | | | | | | | | | | | |
